# Variants in Human Prostacyclin Receptor Gene in Patients with Migraine Headache

**Published:** 2018-10

**Authors:** Fariborz Khorvash, Majid Kheirollahi, Mohammad Kazemi, Gilda Amini, Mehdi Khorrami, Maryam Mirsafaie, Mohammad Reza Mohammadi

**Affiliations:** 1 Department of Neurology, Isfahan University of Medical Sciences, Isfahan, Iran.; 2 Pediatric Inherited Diseases Research Center, Research Institute for Primordial Prevention of Non-Communicable Disease and Department of Genetics and Molecular Biology, School of Medicine, Isfahan University of Medical Sciences, Isfahan, Iran.; 3 Psychiatry and Psychology Research Center, Roozbeh Hospital, Tehran University of Medical Sciences, Tehran, Iran.

**Keywords:** *Iranian Patients*, *Migraine*, *Prostaglandin I2 Receptor Gene*, *Variants*

## Abstract

**Objective:** Prostaglandin I2 receptor plays a major physiologic role in the relaxation of arterial smooth muscle and vasodilation and possibly during migraine attacks. Therefore, in this study, the coding and noncoding exons and exon-intron boundaries of Prostaglandin I2 receptor gene were examined in patients with migraine headache and healthy controls and the potential effects of identified single nucleotide variations were evaluated using direct PCR-sequencing and in silico analysis.

**Method** **:** In this study, the peripheral blood samples of 50 patients and 50 controls were examined to find any mutation in coding and noncoding exons and exon-intron boundaries of PTGIR gene. DNA was extracted and all the samples were amplified by polymerase chain reaction (PCR) and sequenced.

**Results: **In this study, the patients had a mean age of 35.235 ± 10.99 years (range, 9–60 yrs.), and female to male ratio was 4:1 in this group. The controls had a mean age of 35.058 ± 11.116 years (range, 8–59 yrs.), and female to male ratio was 3.7:1.3 in this group. Two patients had mutations in exon 2. The first mutation was located in exon 2 (at amino acid position 251) of PTGIR gene at nucleotide position c.866A > T, a synonymous variant described previously in the database. The second mutation was located in exon 2 c.867G > A, which is a missense variant. Sequence analysis revealed high occurrence of previously reported intronic variants mostly in a homozygous statue.

**Conclusion: **The data supported the hypothesis that mutations in PTGIR gene, particularly the mutation we described, should be considered even in cases of migraine. The presence of this mutation in patients with family history raises important issues regarding genetic counselling.

Migraine is a common chronic or episodic neurological disease characterized by recurrent attack of disabling head pain and associated autonomic nervous system symptoms, such as nausea, vomiting, and sensitivity to light and sound (1, 2). After dental problems and tension type headaches, migraine is the third most common disease. Migraine ia believed to be a multifactorial disorder and, in addition to demographic and environmental factors, genetic predisposition has a significant influence on this disease. 

the basic pathophysiology and precise underlying causes of migraines are not identified (3-5).

The pathophysiology of migraine is complex and considered as a neurovascular disease. Historically, there are two theories for etiology of migraine: vascular and neuronal. Vascular or blood vessel hypothesis of migraine gives importance to cerebral and meningeal arteries and their derangement as a major contributing pathophysiological event. 

According to neuronal hypothesis, which was ﬁrst proposed by Leao, actual changes in blood vessels are a consensus of chemical change in neurons. However, current data demonstrate both neuronal and vascular involvement (3, 6 and 7).

Prostaglandin I2 (PGI2 or prostacyclin) is a member of the eicosanoid family of lipid mediators produced mainly by endothelial cells and plays a role in primary hemostasis by inhibiting platelet activation (8). Also, PGI2 is a potent vasodilator and an important mediator, which acts by activating adenylate cyclase and producing cAMP (9). The release of PGI2 activates and sensitizes meningeal sensory afferents. In this manner, PGI2, produced by endothelial cells, binds to platelet Gs protein‑coupled receptor (prostacyclin receptor) and activates this receptor. Following this event, cyclic adenosine monophosphate is produced and activates protein kinase A. Finally, protein kinase A phosphorylate myosin light chain kinase in smooth muscles, which leads to a decrease its activity. Eventually, this cascade leads to smooth muscle relaxation and vasodilation (10, 11).

In our previous paper, we reported that elevated expression of Prostaglandin I2 receptor (PTGIR) in migraineurs, compared to healthy controls, may have a critical role in triggering migraine attacks (12). The PTGIR gene located on 19q13.3 spans approximately 7.0 kb and is composed of 3 exons that are separated by 2 introns. Encoded protein by this gene is a member of the G-protein coupled receptor family 1 and is a receptor for prostacyclin (prostaglandin I2 or PGI2). Prostaglandin I2 and its receptor plays a major physiologic role in the relaxation of arterial smooth muscle and vasodilation during migraine attacks. Thus, in this study, the direct PCR amplification and sequencing of coding and noncoding exons and exon-intron boundaries of PTGIR gene was examined in patients with migraine headache and healthy controls. Also, the potential effects of identified single nucleotide variations were evaluated using in silico methods to determine the mutations that may be effective in the development of the disease.

## Materials and Methods

The study sample included 50 patients with migraine who referred to local hospitals in Isfahan province (Khorshid hospital). All patients were identified as having a common type of migraine (without aura) using the classification of all headaches, including migraines, which was organized by the International Headache Society and published in the International Classification of Headache Disorders (ICHD) (2). Patients were referred to a neurologist and diagnosed through clinical and paraclinical examinations; then, informed consent was obtained from all of them. 

Peripheral blood samples (2 mL) were collected from the patients and genomic DNA was extracted from the samples according to the manufacturer protocol using PrimePrep Genomic DNA isolation kit (Genet Bio, Korea). Specific primers (Table 1) for 3 exons of PTGIR gene were designed from the intronic regions flanking using Primer3Plus website (http://www.bioinformatics.nl/cgi-bin/primer3plus/primer3plus.cgi) according to the genomic sequence references available at the Genome Browser (http://www.ensemble.org).

The amplification mixture was prepared in 50μL volume including 1X buffer, 1.5 mmol/l magnesium chloride, 200 μmol/l dNTP, 400 nmol/L of each primer, 200 ng/μl DNA, and 2 U Taq DNA polymerase. The PCR reaction was performed under the following conditions: It started with 4 minutes at 94°C and followed by 30 cycles of 30 seconds at 94 °C, 30 seconds at 60°C, 60 seconds at 72°C, and ended after 5 minutes at 72 °C. The PCR products were stained and visualized on a UV transilluminator, following 1.5% gel electrophoresis. The PCR products were sequenced using a cycle sequencing kit on an automated DNA sequencing machine (BigDye Terminator v3.1 and 3730XL DNA analyzer, Applied Biosystems). All sequences were matched to the PTGIR reference sequence using NCBI blast.

This study was approved by the Ethics Committee of Isfahan University of the Medical Sciences and was conducted according to the Declaration of Helsinki. Informed consent was obtained from the patients.

## Results

***Clinical Variables***

This case–control study consisted of 50 patients with migraine and 50 healthy controls. The patients had a mean age of 35.235 ± 10.99 years (range, 9–60 yrs.), and female to male ratio was 4: 1 in this group. The controls had a mean age of 35.058 ± 11.116 years (range, 8–59 yrs.), and female to male ratio was 2.846 in this group.

***PTGIR Gene Mutations***

**Figure 1:**
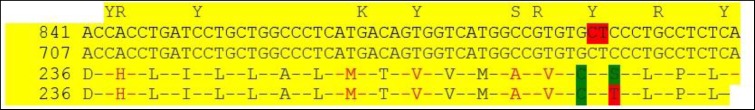
Details of the Sequence Analysis of the PTGIR Gene with Variants from 236 to 255 Amino Acids (Bases 707-766). As It Is Shown, a Missense Mutation Took Place at 252 Amino Acid of Protein

Coding and noncoding exons and exon-intron boundaries of PTGIR gene were sequenced after PCR amplification. PTGIR gene mutations were detected in exons and introns (Table 2, Figure 1). Two patients had mutations in exon 2. The first mutation was located in exon 2 (at amino acid position 251) of PTGIR gene at nucleotide position c.866A > T, which is a synonymous variant and described previously in the database. The second mutation was located in exon 2 c.867G > A, which is a missense variant. This variant results in a serine to threonine substitution at amino acid position 252 in homozygous form (NM_000960.3). This mutation has not been described previously in the database.

Sequence analysis revealed high occurrence of previously reported intronic variants, mostly in a homozygous statue. The variants were distant from exon-intron boundaries or conserved sequences required for splicing. Two missense variants, c.73G>A (p.V25M) and c.867G>A (p.S252M), were found in the second exon of PTGIR, and the second variant was novel ((13)). The variants were in a heterozygous statue and none of them were found in the control group. In silico analysis on p.S252M variant by 3 prediction software highlights its pathogenic effect.

## Discussion

In this study, 8 variants were identified in 17 of 50 patients with the diagnosis of migraine, mostly in a homozygous statue. All the intronic variants were previously reported and listed on dbSNPs, with different allelic frequencies. None of these single nucleotide variations (SNVs) were observed in the controls (100 alleles) who were recruited through the same centers as the patients in Isfahan. In all cases, SNVs rs112967362, rs150178968, rs112167448, rs532147834, rs2229127, and rs14027209 were found only in homozygous state. However, the 2 other variants, rs12459883 and rs11667255, were in both heterozygous and homozygous. Moreover, rs12459883, rs112967362, rs150178968, and rs112167448 had the most frequency. Prostacyclin (PGI2), which acts as a potent vasodilator, is released by vascular endothelial cells. In addition, PGI2 modulates proliferation–migration–differentiation of vascular smooth muscle cells (anti-atherosclerotic) and prevents the aggregation of platelet (anti-thrombotic). The seven transmembrane-spanning G-protein coupled receptor (GPCR), known as the human prostacyclin receptor or hIP, is the recipient of PGI2. Genomic sequencing has revealed numerous SNPs in the PTGIR gene encoding the hIP receptor (14-17). In a study on 9 genetic variations, including 6 SNPs, 1 polymorphism and 2 new variations were found (15).

In this study, some variants were found and comparison of these changes with the available data of previously biochemically characterized mutants revealed a correlation among genetic variants in the hIP receptor, resulting in deficits of hIP receptor function with migraine disease (18, 19). In addition, genetic variants of the hIP receptor may serve as predisposing factors for the outbreak of the disease or as modifying factors of the therapeutic response (14).

Thus, detailed biochemical analysis of genetic variation in functional assays and knowledge of the relationship of desired protein can help find functional correlations of the genetic variances and the disease. In addition, information on the structural requirements of receptor function and performance impairment can help design a drug and develop new therapies for migraine.

## Limitation

Preparing a proper control sample was one of the limitations of this type of studies.

## Conclusion

In conclusion, our data support the hypothesis that mutations in PTGIR gene, particularly the mutation we described, should be considered even in cases of migraine headache. The presence of this mutation in patients with family history of migraine raises the importance of genetic counselling.

**Table 1:** Primer Sets that Were Used for Amplification of PTGIR Exons. Specific Primers for 3 Exons of PTGIR Gene Were Designed from the Intronic Regions Flanking

**Name**	**Sequence**	**Product Size(bp)**
PTGIR-F1 PTGIR-R1	GGGGCAGAGAGAGGAAATGA AATCCGCCATCCCAGGTC	972
PTGIR-F2 PTGIR-R2	AGGGACATCTGAGTGGGCT CCCACGATGTCTCACCTCTT	967
PTGIR-F3 PTGIR-R3	/f	838

**Table 2:** Intronic Variants Found in PTGIR. Sequence Analysis Revealed High Occurrence of Previously Reported Intronic Variants, Mostly in a Homozygous Statue

**Alleles ** **Sample**	**rs11667255**	**rs12459883**	**rs112967362**	**rs150178968**	**rs112167448**	**rs532147834**	**rs140272097**	**rs2229127**
	**G/C**	**C/G**	**C/G**	**A/G**	**G/C**	**T/G**	**G/A**	**C/T**
**Global ** **MAF**	**0.423 (C)**	**0.413 (G)**	**0.027 (G)**	**0.027 (G)**	**0.027 (C)**	**0.012 (G)**	**0.027 (A)**	**0.014 (T)**
15		hetero	homo	homo	homo			
24		hetero	homo	homo	homo			
42		hetero	homo	homo	homo			
47		hetero	homo	homo	homo			
54		hetero	homo	homo	homo			
55		hetero	homo	homo	homo			
66		hetero	homo	homo	homo			
72		homo	homo	homo	homo			
100		homo	homo	homo	homo			
121		homo	homo	homo	homo			
S11			homo	homo	homo			
S19			homo					
S28		homo						
122	hetero					homo	homo	
32	homo					homo	homo	
33	homo					homo	homo	
34	hetero					homo	homo	
32								
33								
34								
64								homo
96								homo
118								homo
119								homo
